# Bacteriological profile and antibiotic susceptibility pattern of septicemia in neonatal intensive care units in Palestine: A retrospective study

**DOI:** 10.1016/j.ijregi.2023.11.019

**Published:** 2023-12-01

**Authors:** Bayan A. Ibrahim, Basma Damiri, Hala Allabadi, Mohammad Qadi

**Affiliations:** 1 Program of Infectious Diseases Prevention and Control, Department of Medical and Health Sciences, Faculty of Graduate Studies, An-Najah National University, Nablus, Palestine; 2 Department of Infectious Diseases, Ettihad Hospital, Nablus, Palestine; 3 Division of Drugs and Toxicology, Department of Biomedical Sciences, Faculty of Medicine and Health Sciences, An-Najah National University, Nablus, State of Palestine; 4 Division of Epidemiology and Public Health, Department of Biomedical Sciences, Faculty of Medicine and Health Sciences, An-Najah National University, Nablus, Palestine; 5 Division of Microbiology, Immunology, and Pathology, Department of Biomedical Sciences, Faculty of Medicine and Health Sciences, An-Najah National University, Nablus, Palestine

**Keywords:** Antibiotic susceptibility, Empiric regimens, Neonatal sepsis, Prevalence rate of NS

## Abstract

**Objectives:**

The aim of this study was to identify the prevalent pathogens that caused neonatal septicemia and their corresponding antimicrobial sensitivities in the neonatal intensive care unit (NICU) in the West Bank.

**Methods:**

In this retrospective study, we extracted records of neonates with suspected neonatal sepsis (NS) admitted to three NICUs in the West Bank between 2019 and 2021 from the Ministry of Health's electronic database.

**Results:**

Out of 6090 suspected episodes of NS, 884 tested positive for blood cultures, and 554 were primary cases; 59.5% were male, 26.6% were preterm, and 52.7% were aged 0-7 days. Moreover, 29% of 884 positive cultures were contributed by multidrug-resistant organisms (MDRO). Only 14.6% of episodes aligned with both Word Health Organization-recommended antibiotic regimens, 5.2% matched with one regimen, and 80.3% did not match either regimen. Specifically, 19.6% of cases matched with ampicillin-gentamicin, and 14.7% matched with ampicillin-cefotaxime. The likelihood of a match between early-onset sepsis and empiric antibiotic regimens was greater than late-onset sepsis (odds ratio = 2.549, [95% confidence interval: 1.276-5.092]). MDRO was more likely than non-MDRO to match with one antibiotic regimen (odds ratio = 4.142 [95% confidence interval: 1.913-8.966]).

**Conclusion:**

There is an alarming prevalence of MDRO among Palestinian neonates in the NICU of the West Bank. The study highlights the urgent need for implementation of effective infection control measures and antimicrobial stewardship programs in the NICUs of hospitals in the West Bank to reduce the prevalence of MDRO and improve neonatal health outcomes.

## Highlights

• High rate of multidrug-resistant organisms in neonatal intensive care units in the West Bank, Palestine.
• World Health Organization empirical regimens may need re-evaluation based on the findings in Palestine.
• Local national protocols and strategies based on antibiograms are necessary.
• Antimicrobial stewardship programs are crucial in preventing multidrug-resistant organism emergence.

## Introduction

Sepsis is a life-threatening condition that can be triggered by microorganisms entering the bloodstream. Neonatal sepsis (NS) can be classified into early-onset sepsis (EOS) and late-onset sepsis (LOS). EOS refers to sepsis that occurs within the first 3 days of birth, while LOS occurs after 3 days of birth [1]. EOS is usually caused by organisms acquired from the mother during delivery or shortly after birth while LOS is typically caused by organisms acquired from the environment, in hospitals or the community [1], [2], [3]. NS remains a major cause of neonatal morbidity and mortality, resulting in 225,000 deaths globally each year despite advances in newborn care [2,4]. When clinical symptoms indicate the possibility of a significant bacterial infection (PSBI), it is crucial to begin antibiotic treatment [5]. As a result, early screenings for neonatal septicemia pathogens, prompt diagnosis, and careful use of antibiotics are crucial for developing effective treatment plans.

Two empiric antibiotic regimens are recommended by World Health Organization (WHO) for treating suspected NS. These are ampicillin-gentamicin, and ampicillin-cefotaxime. Ampicillin is used to treat listeria-causing infections and other gram-positive bacteria like group B *Streptococcus* (GBS), while gentamicin and cefotaxime are used to treat gram-negative bacteria [6]. The matching probability of the WHO empiric antibiotic regimen depends on various factors, including the specific bacterial pathogens that are prevalent in the region, the resistance patterns of these pathogens and the individual patient's clinical presentation and risk factors. The WHO recommends that empiric antibiotic therapy should be based on local epidemiological data and antibiotic susceptibility patterns to maximize the likelihood of selecting an appropriate regimen. They also suggest that regular surveillance of antimicrobial resistance patterns should be conducted to ensure that treatment guidelines are updated accordingly.

In recent years, the emergence of antimicrobial resistance has become a global concern [7]. It is crucial to have an in-depth understanding of the prevalent bacterial pathogens and their antibiotic susceptibility when choosing empirical therapy that can reduce morbidity and mortality [8]. Due to the limited reserve of antibiotics, the increasing antimicrobial resistance poses a significant challenge in managing NS [8].

The neonatal mortality rate in Palestine was 9.3 deaths per 1000 live births in 2021. However, no accurate data is available on neonatal deaths caused by sepsis [9]. The antimicrobial sensitivity of neonatal septicemia in neonatal intensive care unit (NICU) of two government hospitals in Gaza City was studied between 2004 and 2005. It was found that there was a significant presence of multidrug-resistant organisms (MDRO) in neonates with septicemia [10]. The study in Gaza concluded that further national studies are necessary to determine the actual distribution pattern of MDRO in the Palestinian neonatal population to improve therapeutic and treatment options nationwide. Therefore, there is a lack of data on bacterial pathogens and their susceptibility to antibiotics in Palestine. To fill this gap, this study aimed to identify the bacterial profile and sensitivity pattern to neonatal septicemia for each sepsis class (EOS and LOS) in Palestinian neonates aged 0-28 days who were admitted to NICU with suspected NS in three major tertiary governmental hospitals in the West Bank-Palestine from January 2019 to December 2021. The study also aimed to evaluate the matching status of WHO empiric antibiotic regimens with the causative pathogens of NS and determine the risk factors associated with MDRO in the target population. Improving neonatal care, including the prevention, early detection, and treatment of sepsis, is essential for reducing neonatal mortality in Palestine and other countries with high rates of neonatal mortality.

## Methods

### Study design, setting, and population

From January 2019 to December 2021, we collected data on neonates aged 0-28 days who were admitted to NICU with suspected NS in three major tertiary governmental hospitals in the West Bank selected as one hospital for each region, northern region, middle region, and finally southern Palestinian regions, from January, 2019 to the end of December, 2021. The data was obtained retrospectively from the health information system (HIS) of the Palestinian Ministry of Health (MOH), which included all electronic medical records in the years 2019 to 2021.

### Data collection procedure, inclusion, and exclusion criteria

**Figure 1:**
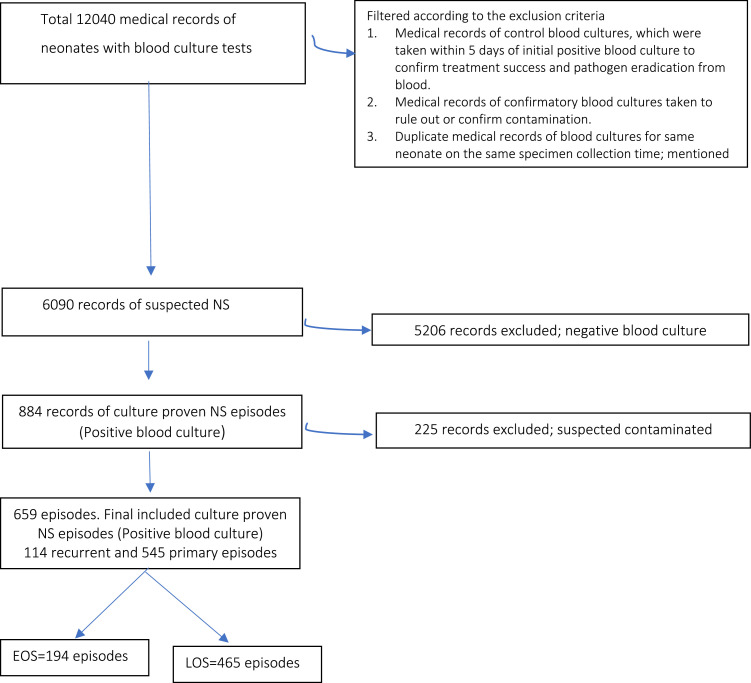
Flow chart explains study procedure for participants selection. Out of the 12,040 records reviewed, 6090 were suspected to be episodes of NS while the remaining 5950 were excluded. Among the 6090 suspected episodes, 884 had positive blood cultures. However, 225 episodes (25.5%) were suspected to have contaminated blood cultures and were therefore excluded. The remaining 659 episodes were categorized into 114 recurrent cases and 545 primary cases. EOS, early onset sepsis; LOS, late onset sepsis; NS, neonatal sepsis.

Overall, we collected a total of 12,040 records. In the case of a new blood culture taken after 5 days from the initial culture, it was classified as a new episode of suspected sepsis. If a neonate medical record showed a positive blood culture for a different pathogen within 48 hours after the initial positive culture, and contamination has been ruled out, it was considered a new episode of NS sepsis case [11]. The excluded medical records consisted of the initial control blood cultures taken within 5 days of the first positive blood culture to verify the effectiveness of treatment and elimination of the pathogen from the blood. Additionally, confirmatory blood cultures were excluded if taken to rule out or confirm contamination. Duplicate medical records of blood cultures for the same neonate at the same time of specimen collection, mentioned as aerobic and anaerobic, were also excluded. The total excluded medical records were 5950; the final number of the studies episodes was 6090. Out of the 6090 suspected NS episodes of NS, 884 episodes (14.5%) had positive blood cultures. However, 225 episodes (25.5%) were suspected of having contaminated blood cultures and were therefore excluded from the study. The remaining 659 episodes were further categorized as 114 recurrent cases and 545 primary cases (Figure 1).

### Operational definition

Suspected NS refers to the condition when a pediatrician orders a blood culture to test for the presence of sepsis-causing organisms. Culture-proven sepsis, on the other hand, is diagnosed when an organism is detected in a blood culture sample. Neonatal infection was divided into two categories based on the age of onset: EOS and LOS. EOS refers to sepsis within the first 3 days of birth, while LOS refers to sepsis after 3 days of birth. MDRO referred to a microbe that has become non-susceptible to at least one agent in three or more antimicrobial categories [12,13].

Matching to one antibiotic regimen: When the blood culture results indicate that the obtained bacteria are sensitive to either gentamicin or cefotaxime, the treatment plan can be matched to the specific antibiotic regimen. On the other hand, when the blood culture results indicate that the obtained bacteria are sensitive to ampicillin or both gentamicin and cefotaxime, the treatment plan can be matched to both antibiotic regimens.

Laboratory tests were conducted to assist in excluding control blood cultures and configuring suspected contaminated blood cultures. These tests included measurements of C-reactive protein levels greater than 9 mg/dl, white blood cell counts less than 4000 × 10^9^ or greater than 20,000 × 10^9^ cells/l, and platelet counts less than 100,000 × 10^9^ cells/l. If a blood culture meets all of the following criteria, there is a suspicion of contamination: The presence of certain bacteria, including coagulase negative *Staphylococcus* (CoNS), *Corynebacterium* species, *Bacillus* species (excluding *B. anthracis*), *Propionibacterium acnes, Propionibacterium* species, *Micrococcus* species, viridians group *streptococci, Aerococcus* species or *Diphtheroid* species, no confirmatory blood culture taken within 5 days of the initial blood culture and negative laboratory signs of infection, such as C-reactive protein, white blood cell, and platelet count. The suspected contaminated blood culture episodes were excluded from the final number of proven culture episodes. In none contaminated blood culture, CoNS were not taken into account for the prevalence calculation of MDRO since their treatment does not rely on sensitivity to the tested drug (Cefoxitin).

As part of our data collection, we also gathered background information such as gender (male, female) and hospital location (northern, middle, southern West Bank) as well as clinical information including postnatal age (0-7 days, 8-28 days) and gestational age (term, preterm).

### Statistical analyses

Chi-squared test was used to estimate the statistically significant difference between categorical variables. Univariate analysis for the association of status matching to whom empiric antibiotics based on other factors was conducted, and crude odds ratio (OR) was used to assess the strength of the association with a 95% confidence interval (CI). Adjusted logistic regression analysis models were conducted to determine the association between MDRO (yes, no as a reference group) and sepsis class (EOS, LOS as a reference group). The model was adjusted to gender (female, male as a reference group), gestational age (term, preterm as a reference group), gram stain (negative, positive as a reference group), location of the hospital (northern, southern, and middle West Bank as a reference group), year (2019, 2020, 2021 as a reference group), postnatal age (0-7 days, 8-28 days as a reference group), mortality (lived, died as a reference group), ampicillin-gentamicin matching status to WHO empiric antibiotic regimen (match, did not match as a reference group), and ampicillin-cefotaxime matching status to WHO empiric antibiotic regimen (match, did not match as a reference group). Adjusted odds ratios and their 95% CI were used as indicators of levels of association. The analyses of the results also proceeded with the use of the Statistics Package for Social Sciences (SPSS. Version 22). *P*-values <0.05 were considered statistically significant.

### Ethical approval

Ethical approval (Ref. Mas. Dec 2021/9) was obtained from the Institutional Review Board (IRB) at An-Najah National University in Palestine. Additional approval to access medical fields from the Palestinian Ministry of Health was obtained. All data were collected and treated confidentially, kept safe, and available only for the researchers. Personal and medical information was collected and analyzed anonymously. Codes were used instead of names to ensure confidentiality.

## Results

There were 659 episodes, 114 being recurrent and 545 being primary cases; 59.4% were males, 26.6% were preterm neonates, and 52.7% were 0-7 days old. The live births reached 44,625 during the same period, and therefore, the prevalence rate of culture-proven NS was 12 per 1000 live births (Table 1).

**Table 1:** Background information of neonates with culture-proven sepsis admitted to the neonatal intensive care unit (n = 545 primary cases).

Variable	Category	n (%)
Gender	Male	324(59.4)
	Female	221(40.6)
Gestational age	Term	460(73.4)
	Preterm	145(26.6)
Mortality	Lived	423(77.6)
	Died	122(22.4)
Year	2019	173(31.7)
	2020	182(33.4)
	2021	190(34.9)
Hospital	Middle region hospital	102(18.7)
	Southern region hospital	125(22.9)
	Northern region hospital	318(58.3)
Postnatal age	0-7 days old	287(52.7)
	8-28 days old	258(47.3)
Age per days	Median age (interquartile range)	7(13)

The final number of episodes was 659; 2.4% showed the presence of yeast, and 63.5% were tested with gram-positive bacteria. The most prevalent pathogens were CoNS (49.2%), *Klebsiella* species (18.1%), and *Streptococcus* species (7.9%). The most prevalent organism in suspected contaminated blood culture was CoNS (78.2%), followed by *Streptococcus* species (15.6%) (Table 2-Part A). Most (70.6%) were classified as LOS, while EOS accounted for 29.4%. Additionally, 29% of all cultures were contributed by MDRO. The study found that a significant proportion of gram-positive bacteria were present in both EOS NS at 72.9% and LOS NS at 61.7% (*P*-value = 0.006). Only 14.6% aligned with both WHO-recommended antibiotic regimens, while the majority (80.3%) did not match either. Furthermore, 19.6% of cases matched with ampicillin-gentamicin, and 14.7% matched with ampicillin-cefotaxime. Antibiotic susceptibility for gram-negative bacteria was high for amikacin (63%), meropenem (70%), piperacillin-tazobactam (65.6%), and colistin (100%). However, it was low for ampicillin (7.1%), cefotaxime (21.8%), and ceftazidime (29%). On the other hand, gram-positive bacteria were sensitive to vancomycin (99.8%), and 81.4% of gram-positive organisms other than *Staphylococcus* spp. were sensitive to ampicillin (see Table 2- Part B).

**Table 2:** Bacteriological profile of neonates with culture proven sepsis admitted to the neonatal intensive care unit (n = 659 episodes).

Part A - Bacteriological profile of neonates with culture proven sepsis admitted to the neonatal intensive care unit (n = 659 episodes)				
Organism name	Category	n (%)	MDRO n(%)	Mortality n(%)
Gram-positive bacteria	Coagulase-negative *staphylococci*	324(49.2)	224(69.1)	58 (18)
	*Staphylococcus aureus*	38(5.8)	27(71)	8(21)
	*Streptococcus* spp*.*	52(7.9)	9(17.3)	8(15.3)
	*Bacillus* spp*.*	3(0.5)	0(0)	0(0)
	*Micrococcus* spp*.*	2(10.3)	0(0)	0(0)
	Total	419(63.5)	260(62)	72(17.2)
Gram-negative bacteria	*Klebsiella* spp*.*	119(18.1)	104(87.4)	33(27.7)
	*Escherichia coli*	42(6.4)	22(52.2)	15(35.7)
	*Pseudomonas* spp*.*	11(1.7)	1(9.1)	1(9)
	*Acinetobacter* spp*.*	29(4.4)	23(79.3)	10 (34.5)
	*Stenotrophomonas* spp	11(1.7)	11(100)	2(182)
	*Enterobacter* spp*.*	4(0.6)	0(0)	0(0)
	*Serratia* spp	4(0.6)	2(50)	2(50)
	*Hemophillus influenza*	4(0.6)	0(0)	0(0)
	Total	224(34)	163	61(27.2)
Fungus	**Yeast**	16(2.4)	0(0)	1(6.2)


Part B - Characteristics for clinical variables of the neonates with culture-proven sepsis (n = 659 episodes)				
Sepsis class	EOS ≤ 72 hours of life	194(29.4)		
	LOS> 72 hours of life	465(70.6)		
Gram stain	Gram-positive EOS	140(72.9		
	Gram-negative EOS	52(27.1)		
	Gram-positive LOS	279(61.7		
	Gram-negative LOS	173(38.3)		
MDRO	Yes	191(29.0)		
	No	468(71.0)		
Resistance pattern	Extended Spectrum Beta Lactamase	93(14.1)		
	Carbapenem Resistant Enterobacteriaceae	35(5.3)		
	Methicillin Resistant Staphylococcus Aureus	26(3.9)		
	*Acinetobacter baumannii*	23(3.5)		
	Vancomycin Resistant Enterococcus	1(0.2)		
	*Pseudomonas* MDRO	1(0.2)		
General matching status to WHO empiric antibiotics regimens.	Matched with both regimens.	96(14.6)		
	Matched to one regimen.	34(5.2)		
	Matched to non-regimen.	529(80.3)		
Specific matching status to WHO empiric antibiotics regimens.	Matched to ampicillin-gentamicin.	129(19.6)		
	Matched to ampicillin-cefotaxiem.	97(14.7)		

EOS, early onset sepsis; LOS, late onset sepsis; MDRO, multi drug resistant organism; WHO, World Health Organization.

The study revealed significant variations in matching status, gram stain, MDRO, sepsis class, and resistant pattern (*P*-value<0.001), postnatal age (*P*-value = 0.033), and gestational age (*P*-value = 0.0036). Most gram-positive bacteria (88.3%) did not correspond to any treatment regimen. Almost half (49%) of the bacteria that matched both treatment regimens were identified as gram-positive. In comparison, most bacteria matched with only one regimen were gram-negative (94.1%). Only 0.5% of the MDRO matched both regimens, while 11.5% matched one regimen, and 88.0% did not match any regimen. The study also found that 18.1% of neonates aged 0-7 days and 11% of neonates aged 8-28 days matched with both empiric antibiotics regimes (*P*-value = 0.033). Most of the EOS cases were among those who matched for both regimens (53.2%), while most of the cases that matched for one regimen were LOS cases (71.8%) (*P*-value < 0.001). Moreover, most deaths (80.9%) were associated with bacteria that did not match any treatment regimens (Table 3).

**Table 3:** Status matching to WHO empiric antibiotics based on general and clinical factors.

Variable	Category	Matched to both regimen n (%)	Matched to one regimen n (%)	Matched to non-regimen n (%)	*P*-value
Gender	Female	35(13.1)	19(7.1)	213(79.8)	0.139
	Male	61(63.5)	15(3.8)	316(80.6)	
Postnatal age	0-7	60(18.1)	16(4.8)	256(77.1)	0.033
	8-28	36(11)	18(5.5)	273(83.5)	
Gram stain	Negative	49(21.8)	32(14.2)	144(64)	<0.001
	Positive	47(11.2)	2(0.5)	370(88.3)	
Multi drug resistant organism	Yes	1(0.5)	22(11.5)	168(88)	<0.001
	No	95(20.3)	12(2.6)	361(77.1)	
Sepsis class	Early onset sepsis	47(24.2)	8(4.1)	139(71.6)	<0.001
	Late onset sepsis	49(10.5)	26(5.6)	390(83.9)	
Mortality	Lived	76(14.5)	28(5.4)	419(80.1)	0.929
	Died	20(14.7)	6(4.4)	110(80.9)	
Gestational age	Term	70(14.7)	18(3.8)	389(81.6)	0.036
	Preterm	26(14.3)	16(8.8)	140(76.9)	
Hospital location in the West Bank	Northern	47(12.4)	16(4.2)	316(83.4)	0.191
	Southern	24(16.1)	9(6)	116(77.9)	
	Middle	25(19.1)	9(6.9)	97(74)	
Year	2019	31(15.5)	12(6)	157(78.5)	0.614
	2020	31(14)	14(6.3)	177(79.7)	
	2021	34(14.3)	8(3.4)	195(82.3)	

Matching to one antibiotic regimen: When the blood culture results indicate that the obtained bacteria are sensitive to either gentamicin or cefotaxime, the treatment plan can be matched to the specific antibiotic regimen. On the other hand, when the blood culture results indicate that the obtained bacteria are sensitive to ampicillin or both gentamicin and cefotaxime, the treatment plan can be matched to both antibiotic regimens.

In Supplementary File 1, findings from the crude analysis for MDRO prevalence and other factors showed that certain factors negatively correlate with NS blood culture with MDRO compared to non-MDRO. These factors include postnatal age (0-7 days) compared to (8-28 days) (OR = 0.656, *P*-value = 0.016), sepsis class EOS compared to LOS (OR = 0.380, *P*-value <0.001), survival outcome lived compared to died (OR = 0.561, *P*-value = 0.004), and matching status, precisely matched compared to non-matched for ampicillin-gentamicin (OR = 0.468, *P*-value = 0.002) and ampicillin-cefotaxime (OR = 0.020, *P*-value <0.001). Furthermore, gram-negative stains were more likely to be MDRO compared to gram-positive stains (OR = 38.17, *P*-value <0.001) (Supplementary file 1).

We conducted an adjusted binary logistic regression analysis to investigate the association between MDRO and sepsis class. The analysis showed that EOS had a 66.1% lower chance of having a blood culture with MDRO than LOS (OR = 0.339, *P*-value = 0.048). Additionally, gram-negative bacteria were found to be 1097 times more likely than gram-positive bacteria to be MDRO (OR = 1097.7, *P*-value <0.001). Furthermore, the presence of MDRO in blood culture was 95.5% less likely to match to ampicillin-gentamicin regimen (OR = 0.045, *P*-value <0.001), and 99.8% less likely to match to ampicillin-cefotaxime regimen (OR = 0.002, *P*-value <0.001). We also found that hospitals in the southern West Bank region were less likely to have MDRO than those in the central region (OR = 0.123, *P*-value <0.001). Finally, it was more likely to encounter MDRO in the year 2020 than in 2021 (OR = 2.683, *P*-value <0.001) (Table 4).

**Table 4:** Adjusted binary logistic regression for the association between MDRO and sepsis classes.

MDRO (Yes)^a^	Covariate category	Covariate reference	Odds ratio	95% confidence interval	*P*-value
Gender	Female	Males	0.999	0.484-2.062	0.997
Gestational age	Term	Preterm	0.707	0.258-1.936	0.500
Gram stain	Negative	Positive	1097.7	322.9-3732.5	<0.001
Sepsis class	Early onset sepsis	Late onset sepsis	0.339	0.116-0.992	0.048
Location	Northern	Middle	0.608	0.201-1.843	0.379
	Southern		0.123	0.0470-0.323	<0.001
Year	2019	2021	1.657	0.625-4.399	0.310
	2020		2.683	1.046-6.882	0.040
Postnatal age	0-7 days	8-28 days	1.309	0.567-3.002	0.528
Mortality	Lived	Died	0.582	0.251-1.350	0.207
Ampicillin-Gentamicin matching status to WHO empiric antibiotic regimen	Matched	Not matched	0.045	0.012-0.170	<0.001
Ampicillin-Cefotaxime matching status to WHO empiric antibiotic regimen	Matched	Not matched	0.002	0.000-0.024	<0.001

Abbreviations: MDRO: multi drug resistant; WHO: World Health Organization.

a The reference category for MDRO is No.

## Discussion

Neonatal sepsis is a serious health concern that affects newborns within the first month of their lives, resulting in high neonatal morbidity and mortality rates. Early screenings, prompt diagnosis, and responsible use of antibiotics are essential to ensure effective treatment, particularly in low- and middle-income countries. However, the limited supply of antibiotics due to antimicrobial resistance poses a significant challenge. This worldwide issue demands urgent attention. When selecting the right empirical therapy to reduce morbidity and mortality, it is essential to understand the common bacterial pathogens and their susceptibility to antibiotics [8]. Unfortunately, Palestine lacks essential information on prevalent bacterial pathogens and their antibiotic susceptibility patterns, hampering efforts to decrease morbidity and mortality rates. This study aimed to present an evaluation of NS that looks at how well WHO antibiotic regimens match local bacterial sensitivity patterns and resistance factors.

The results of this study have several important implications for clinical practice. Only 14.6% of the NS organisms confirmed through culture matched both of the WHO empiric regimens, and 5.2% matched one of them. In contrast, most of the organisms did not align with any empiric antibiotic regimens, consistent with other studies [12]. Previous research showed that gentamicin is more effective than cefotaxime in treating non-meningitis-related NS due to its comprehensive coverage and lower mortality rate [6]. This study revealed that the success rate of a match with ampicillin-gentamicin was only 19.6%, whereas ampicillin-cefotaxime had a 14.7% chance of matching with the organism. Although previous research has favored gentamicin over cefotaxime for NS, our study found that the success rate of ampicillin-gentamicin was relatively low compared to studies conducted in nearby countries such as Egypt and Jordan [2,12]. One possible explanation for this variation could be the timing of the studies. Our study is more recent, and it is known that with time, resistance tends to increase [14]. Differences in clinical practice and socioeconomic factors between different areas may also have an impact.

According to a study in Egypt, only 9.2% of the cases tested positive for MDRO [15]. This study found that one-third of cultures tested positive for MDRO, indicating a lack of antimicrobial stewardship programs and inadequate implementation of infection control policies [16,17]. Blood cultures with MDRO were less likely to align with antibiotic treatments, linking MDRO with extended hospital stays and gram-negative bacteria.

It is crucial to base treatment on institutional antibiograms, especially for patients with LOS [6]. The results of this study align with previous research, demonstrating a link between the occurrence of MDRO and both LOS and the presence of gram-negative bacteria [18,19]. The higher prevalence of MDRO among neonates LOS and late postnatal age is believed to be due to the transmission of antibiotic-resistant bacteria within hospital settings, which are significantly impacted by infection prevention measures and antibiotic exposure [18,19]. The results also are consistent with global studies that show a higher incidence of MDRO in late infections arising from the environment around neonates in community or hospital settings [6,11,15,20,21]. A recent study in Gaza found that changing the treatment for neonatal sepsis to vancomycin-meropenem, based on the specific antibiogram of the hospital, could be beneficial [10]. However, more local research on the West Bank needs to be conducted, making it difficult to assess the situation there. It is crucial to implement a national plan to ensure adequate neonatal care, especially in the southern region, to decrease the rates of neonatal morbidity and mortality. It has been observed that hospitals in the southern region have a higher prevalence of MDRO and a higher mortality rate for neonatal sepsis compared to the middle region in this study, while matching to both regimens was less likely to be prevalent in the northern region than the middle. Further research is needed to determine the reasons behind this trend. This highlights the importance of a nationwide approach to selecting the appropriate treatment based on the unique antibiogram of each hospital.

Neonatal mortality is a critical public health issue in Palestine. Factors contributing to neonatal mortality in Palestine include inadequate access to healthcare services and resources, poor maternal health, and political instability and conflict. Neonatal sepsis is a leading cause of death in newborns, particularly in low-resource settings [22,23]. Neonatal mortality due to sepsis is a concerning issue in Palestine. The mortality rate for NS cases in this study was 22.4%, which exceeds the global mortality range of 11-19% among nine high-income countries and three low-middle-income countries [24]. Furthermore, most deaths (85.7%) were caused by bacteria that did not match either treatment regimen. Regrettably, the information available regarding mortality is limited to infants diagnosed with culture-positive NS who had already passed away by the time the culture results were communicated. Therefore, we cannot definitively determine sepsis as the sole contributor to mortality. Additionally, we do not have any data on individuals who passed away after receiving the culture results. Thus, we suggest further research to explore the root causes of the elevated mortality rate among Palestinian newborns. Consistent with previous studies, neonates with MDRO-related sepsis exhibit a greater likelihood of mortality [25,26]. This highlights the urgent need for improved maternal and neonatal healthcare services in Palestine, including better prenatal care, access to skilled birth attendants, and improved infection prevention and control measures in healthcare facilities. Addressing the issue of neonatal sepsis requires a comprehensive approach that involves educating the community about the importance of proper hygiene practices, improving access to healthcare services, and strengthening the healthcare system to ensure that all newborns receive the care they need to survive and thrive.

The findings indicated that CoNS, *Klebsiella*, and *Streptococcus* were the most common pathogens in all episodes. This was consistent with a study conducted in Gaza that found these organisms and *Escherichia coli* [10]. It is natural for CoNS to be present on human skin [13]. Moreover, CoNS sepsis frequently occurs in the NICU due to prevalent molecular strains among infants and healthcare personnel [27,28]. Certain strains may persist in the NICU for years. Therefore, the percentage of CoNS in this study was overestimated, as 35.1% of CoNS episodes were identified as contaminated blood cultures. Despite their low virulence, these organisms can potentially cause significant infections in the bloodstream and other areas of the body [27]. Moreover, they are the significant causative microorganisms in neonatal nosocomial sepsis [28]. It was also concluded that neonatal CoNS sepsis was increasingly caused by a limited number of predominant molecular CoNS types and that antibiotic resistance is probably a major selective force [28]. It should be noted that although 69.1% of CoNS were found to be resistant to cefoxitin, all showed sensitivity to vancomycin. Nevertheless, the growing prevalence of MDR CoNS in NICUs in the West Bank suggests that they are potential health risks.

In 2019-2021, there were 12 cases of culture-proven NS per 1000 live births. This rate is relatively higher than that of high-income countries [16] but close to the results observed in Egypt [15] and India [21]. These findings highlight the significant role of clinical procedures and socioeconomic factors in the incidence of neonatal sepsis [17]. Moreover, the contamination rate in the NICU was higher than the accepted rate of 2-3% in blood culture [24] and the contamination rate in the Gaza study [6]. However, it was similar to another study that observed a contamination rate of 16.4% due to improper sample collection, handling of microbiological specimens, and poor environmental or personal hygiene practices during culturing using clean but not sterile techniques [29]. This suggests that the quality of clinical pictures and adherence to contamination-free measures may need to be improved [17]. To prevent the spread of resistant organisms in NICU, it is crucial to identify the causes of multi-drug resistance in isolated bacteria. This may involve investigating factors like antibiotic overuse, suboptimal infection control practices, and inadequate prescribing habits among healthcare providers. Such information can help develop targeted interventions to tackle this issue effectively. Assessing Palestine's healthcare system and infrastructure is crucial as it may affect the incidence and treatment of neonatal sepsis. This evaluation should consider resource availability, staffing, and funding for infection control initiatives.

This study has some potential limitations. First, the inability to access data from private hospitals, which may have different bacteriological profiles and susceptibility patterns of neonatal sepsis depending on infection control policies and antibiotic use practices. Additionally, the study was conducted during the COVID-19 pandemic. However, our data showed no significant differences between the three studied years regarding the number of cases and the matching status with the WHO empiric antibiotic regimens for treating suspected NS, except for MDRO-related sepsis in 2020 compared to 2021. Therefore, further studies are recommended after the pandemic crisis, as the results may vary. Despite these limitations, this study has important clinical implications as it is the first evaluation of NS and WHO antibiotic regimens match local bacterial sensitivity patterns and resistance factors. This groundbreaking study used a comprehensive and representative research sample to provide valuable insights into the prevalence and various types of multidrug-resistant bacteria found in NICUs in the West Bank. Its findings can serve as a valuable reference for decision-makers seeking to implement effective measures and assess their effectiveness in the future.

## Conclusion

The results of a recent study highlight the alarming prevalence of MDRO among Palestinian neonates in the NICU of the West Bank. Institution-specific protocols and a national strategy for suitable treatments are needed. Antimicrobial stewardship programs should be implemented to prevent the emergence of MDRO. Further research is recommended to explore the causes of drug inefficacy.

## Declaration of Competing Interest

The authors have no competing interests to declare.

## Appendix Supplementary materials

Image, application 1
