# Supplementary material for: Bacteriological profile and antibiotic susceptibility pattern of septicemia in neonatal intensive care units in Palestine: A retrospective study
Source: IJID Reg. 2023 Dec 1;10:87–93. doi: 10.1016/j.ijregi.2023.11.019 (PMC10764240; doi:10.1016/j.ijregi.2023.11.019)
Supplement: Supplementary file 1 [file mmc1.docx]

In Supplementary file 1, findings from the crude analysis for MDRO prevalence and other factors showed that certain factors have a negative correlation with NS blood culture with MDRO compared to non-MDRO. These factors include postnatal age (0-7 days) compared to (8-28 days) [OR=0.656, P-value=0.016), sepsis class EOS compared to LOS [OR=0.380, P-value<0.001], survival outcome lived compared to died [OR=0.561, P-value=0.004], and matching status, specifically matched compared to non-matched for Ampicillin-Gentamicin [OR=0.468, P-value=0.002] and Ampicillin-Cefotaxime [OR=0.020, P-value<0.001]. Furthermore, Gram-negative stains were more likely to be MDRO compared to Gram-positive stains [OR=38.17, P-value<0.001] (Supplementary file 1).

**Supplementary file 1: Univariate analysis for MDRO prevalence and other general and clinical factors**

|  |  | MDRO n (%) | |  |  |  |
| --- | --- | --- | --- | --- | --- | --- |
| Variable | Category | Yes | No | Odds Ratio | 95% CI | P value |
| Gender | Female | 82(42.9) | 185(39.5) | 1.151 | 0.818-1.619 | 0.432 |
|  | Male | 109(57.1) | 283(60.5) |  |  |  |
| Postnatal age | 0-7 | 82(42.9) | 250(53.4) | 0.656 | 0.467-0.921 | 0.016 |
|  | 8-28 | 109(57.1) | 218(46.6) |  |  |  |
| Gram stain | Negative | 163(85.8) | 82(13.7) | 38.170 | 23.444-62.146 | <0.001 |
|  | Positive | 27(14.2) | 392(86.3) |  |  |  |
| Sepsis class | EOS | 32(16.8) | 162(34.6) | 0.380 | 0.249-0.581 | <0.001 |
|  | LOS | 159(83.2) | 306(65.4) |  |  |  |
| Mortality | Lived | 138(72.3) | 385(82.3) | 0.561 | 0.378-0.834 | 0.004 |
|  | Died | 53(27.7) | 83(17.7) |  |  |  |
| Gestational age | Term | 125(65.4) | 352(75.2) | 0.624 | 0.433-0.899 | 0.013 |
|  | Preterm | 66(34.6) | 116(24.8) |  |  |  |
| Ampicillin-Gentamicin matching status | Matched | 23(12) | 106(22.6) | 0.468 | 0.287-0.761 | 0.002 |
|  | Not matched | 168(88) | 362(77.4) |  |  |  |
| Ampicillin-Cefotaxime matching status | Matched | 1(0.5) | 96(20.5) | 0.020 | 0.003-0.147 | <0.001 |
|  | Not matched | 109(99.5) | 372(79.5) |  |  |  |
| Hospital location in the West Bank | Northern | 116(60.7) | 263(56.2) |  |  | 0.108 |
|  | Southern | 33(17.3) | 116(24.8) |  |  |  |
|  | Middle | 42(22) | 89(19) |  |  |  |
| Year | 2019 | 57(29.8) | 143(30.6) |  |  | 0.243 |
|  | 2020 | 73(38.2) | 149(31.8) |  |  |  |
|  | 2021 | 61(31.9) | 176(37.6) |  |  |  |
